# Evaluation of Merkel Cell Polyomavirus DNA in Tissue Samples from Italian Patients with Diagnosis of MCC

**DOI:** 10.3390/v13010061

**Published:** 2021-01-05

**Authors:** Carla Prezioso, Raffaella Carletti, Francisco Obregon, Francesca Piacentini, Anna Maria Manicone, Giuseppe Soda, Ugo Moens, Cira Di Gioia, Valeria Pietropaolo

**Affiliations:** 1 Department of Public Health and Infectious Diseases, Sapienza University of Rome, 00185 Rome, Italy; carla.prezioso@uniroma1.it (C.P.); francisco.obregon1703@gmail.com (F.O.); piacentini.1854105@studenti.uniroma1.it (F.P.); 2 Microbiology of Chronic Neuro-degenerative Pathologies, IRCSS San Raffaele Pisana, 00163 Rome, Italy; 3 Department of Translational and Precision Medicine, Sapienza University of Rome, 00185 Rome, Italy; affaella.carletti@uniroma1.it; 4 Division of Pathology, “S.M. Goretti” Hospital, 04100 Latina, Italy; annamaria.manicone@uniroma1.it; 5 Department of Molecular Medicine, Sapienza University of Rome, 00161 Rome, Italy; giuseppe.soda@uniroma1.it; 6 Department of Medical Biology, Faculty of Health Sciences, University of Tromsø, The Arctic University of Norway, 9037 Tromsø, Norway; ugo.moens@uit.no; 7 Department of Radiological, Oncological and Pathological Sciences, Sapienza University of Rome, 00161 Rome, Italy; cira.digioia@uniroma1.it

**Keywords:** Merkel cell polyomavirus, MCC diagnosis, primary lesions, metastatic lesions, GTTGA insertion, hit-and-run

## Abstract

Because the incidence of Merkel cell carcinoma (MCC) has increased significantly during the last 10 years and it is recognized that Merkel cell polyomavirus (MCPyV) and ultraviolet (UV) radiation represent two different etiological inputs sharing clinical, histopathological, and prognostic similar features, although with different prognosis, this study investigated the detection of MCPyV in skin and lymph nodes with histological diagnosis of MCC. Formalin-fixed paraffin-embedded tissue (FFPE) were retrieved from archived specimens and MCPyV non-coding control region (NCCR) and viral capsid protein 1 (VP1) sequences were amplified and sequenced. Results provide an interesting observation concerning the discrepancy between the MCPyV DNA status in primary and metastatic sites: in fact, in all cases in which primary and metastatic lesions were investigated, MCPyV DNA was detected only in the primary lesions. Our data further support the “hit-and-run” theory, also proposed by other authors, and may lead to speculation that in some MCCs the virus is only necessary for the process of tumor initiation and that further mutations may render the tumor independent from the virus. Few point mutations were detected in the NCCR and only silent mutations were observed in the VP1 sequence compared to the MCPyV MCC350 isolate. To unequivocally establish a role of MCPyV in malignancies, additional well-controlled investigations are required, and larger cohorts should be examined.

## 1. Introduction

Merkel cell carcinoma (MCC) is a rare and highly aggressive neuroendocrine skin cancer. Accumulating evidence suggests that MCC pathogenesis could be associated with the presence of Merkel cell polyomavirus (MCPyV), a small non-enveloped DNA virus, characterized by a circular double-stranded genome encompassing three functional domains, a non-coding control region (NCCR), and the early and late regions. The NCCR contains the viral origin (Ori) of replication and bidirectional promoters for viral transcription of the early and late regions [1]. The late region encodes for two capsid proteins, virus protein 1 (VP1) and virus protein 2 (VP2) [1,2]. The early region contains the “Tumor” (T) antigen gene locus, from which alternatively spliced RNA transcripts are produced. This region encodes for the large T (LT), small (sT), 57kT antigens and for a product from an alternate frame of the LT open reading frame (ALTO) [1]. The MCPyV LT antigen contains motifs and domains, playing a prominent role in viral genome replication, transcription and tumorigenesis [1]. sT also contributes to tumorigenesis, whereas the functions of 57kT and ALTO remain obscure. To date, the link between MCPyV infection and cell transformation still needs to be clarified, although it has been established that viral DNA integration into the host genome and expression of the C-terminal truncated LT are required for MCC development [3]. The C-terminus of LT contains anti-tumorigenic properties and may explain why this region is deleted in MCC [4]. 

From the first study by Feng et al. [5] to date, it has been established that ~80% of MCC harbored MCPyV genome clonally integrated [2]. Before the large use of CK20 immunostaining, the pathology diagnosis was difficult and required electronic microscopy, thus, true MCC were frequently misclassified [6,7]. Today, immunohistochemistry is frequently performed to confirm the MCC diagnosis using a combination of neurofilament, cytokeratin 20 (CK20), CK7, and thyroid transcription factor-1 stains characterized by a high sensitivity and specificity in distinguishing MCC [8,9,10,11]. MCC can also occur in association with UV radiation–induced alterations involving mutations, heterozygous deletion, and hypermethylation of the *Retinoblastoma* gene [12]. MCPyV and UV represent two different etiological inputs sharing clinical, histopathological, and prognostic similar features, although with different prognosis [13]. Recently it has been reported that MCPyV-positive MCCs show less metastatic tendency and better prognosis than MCPyV-negative MCCs [13]. Given the potential prognostic differences between these two tumor types, our study was aimed to investigate the detection of MCPyV and viral expression in tissue samples from Italian patients diagnosed with MCC. 

## 2. Materials and Methods 

### 2.1. Clinical Specimens

Formalin-fixed paraffin-embedded tissue (FFPE) of skin and lymph nodes with histological diagnosis of MCC retrieved from the archived specimens of the Division of Pathology of both Policlinico Umberto I—Sapienza University of Rome (Rome) and S.M. Goretti Hospital (Latina), were obtained from 26 patients undergoing surgery (12 males and 14 females, age range 74–96 years, mean age 79.5 ± 6.8 years) from April 2005 to August 2020. Out of all 26 analyzed specimens (MCC 1–26), 17 (MCCPL 1–17) were MCC primary lesions (skin) and 9 (MCCML 1–9) metastatic lesions (lymph node) (Table 1).

**Figure 1:**
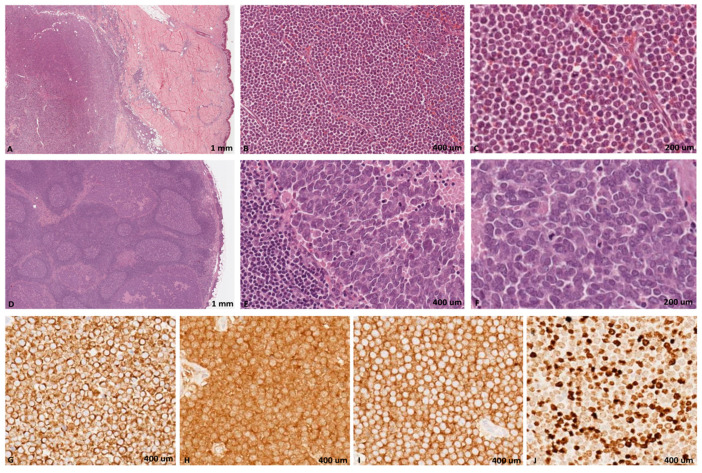
Merkel cell carcinoma. A malignant blue cell tumor nodule in dermis and adipose tissue of subcutis (**A**) and lymph node epithelial metastasis (**D**). Both in subcutaneous (**B**,**C**) and lymph nodal (**E**,**F**) sides, there is a proliferation of intermediate round tumor cells with fine granular chromatin and nuclear molding (**H**,**E**,**A**,**D** 5×; **B**,**E** 20×; **C**,**F** 40×). The tumor cells are positivity for CK20 (**G**), synaptophysin (**H**) and CD56 (**I**), with high proliferation index (**J**, Ki67 of about 70%) (**G**–**J** immunohistochemical staining, 40×).

The histological diagnosis was confirmed by all the pathologists based on light microscopy examination of hematoxylin-eosin (H&E) and immunostained sections. Immunohistochemical stains were performed by BOND-III automated IHC stainer (Leica Biosystems, Milan, Italy) with the following BOND ready-to-use antibodies (Novocastra, Newcastle upon Tyne, UK): cytokeratin 20, synaptophysin, CD56, and Ki67, using HRP-DAB detection system. Microscopically, in both the cutaneous and lymph node side, the neoplasia was characterized by a proliferation of round cells with intermediate size, chromatin with fine granular pattern, nuclear molding, and numerous mitotic figures. The neoplastic cells showed positivity for the epithelial marker CK20 with perinuclear dot-like pattern and for neuroendocrine markers synaptophysin and CD56. In all cases the proliferation index evaluated by Ki67 antibody was high (mean value 85%) (Figure 1).

This research study was conducted retrospectively from data obtained for clinical purposes. We consulted extensively with the Ethic Committee Sapienza University of Rome, Policlinico Umberto I who determined that our study did not need ethical approval.

### 2.2. DNA Extraction

Following deparaffinization with xylene, total DNA was extracted from FFPE by QIAamp^®^ DNA FFPE Tissue Kit (QIAGEN, S.p.A, Milano, Italy) according to the manufacturer’s instructions. The extracted nucleic acids were eluted in a final volume of 50 μL and DNA was evaluated for its PCR suitability by amplifying the β-globin gene sequences [14].

### 2.3. Real-Time Polymerase Chain Reaction (qPCR)

The presence and quantity of viral DNA in FFPE sections were carried out by quantitative polymerase chain reaction (qPCR) after DNA extraction, using primer and probe, targeting sT *gene*, as previously described [15]. 

### 2.4. MCPyV Nested PCR 

Positive MCPyV DNA samples were subjected to nested PCR with different MCPyV-specific primer pairs mapping VP1 and NCCR regions of the genome and subsequently sequenced, following published protocols [5,16,17].

### 2.5. MCPyV Phylogenetic Analysis

A phylogenetic tree was generated using Molecular Evolutionary Genetics Analysis (Mega) version 6.0 software program [18] after aligning the VP1 sequences isolated from samples to those of the reference MCPyV isolate MCC350 (EU375803) [5]. A bootstrap test with 1000 replicates was performed to evaluate the confidence of the branching pattern of the tree. 

### 2.6. Statistical Analysis

MCPyV detection was analyzed by counts and proportions. Continuous variables normally distributed were expressed as mean ± SD. Continuous variables not normally distributed were expressed by median and range. To evaluate differences for categorical variables, χ^2^ test was performed.

## 3. Results and Discussion

MCPyV DNA was detected in 13/26 samples (50%) (*MCC 1–13*). All were primary lesions (13/17, 76.5%) and presented, by qPCR, an average value of viral DNA of 70 × 10^−1^ copies/μg (95% CI 69–72). Five out of 13 MCCPL (38.5%) were head and neck (H&N) carcinomas (*MCCPL 9–13*) and 8/13 (61.5%) were in the skin of the lower limb and of arm/forearm/shoulder (*non*-H&N) (*MCCPL 1–8*). An average value of viral DNA of 63 × 10^−1^ copies/μg (95% CI 62–65) was detected among H&N carcinomas and of 74 × 10**^−^**^1^ copies/μg (95% CI 73–76) among tumors *non-*H&N. The remaining 4 of 17 MCCPL (*MCCPL 14–17*) did not revealed MCPyV DNA, nor could MCPyV DNA be amplified from any of the 9 MCCML samples *(MCC 18–26)* (Table 1).

Modifications in VP1 and NCCR sequencing with relative genotype were reported in Table 2.

The relationship between MCPyV-positive and -negative cases and worse or favorable outcome was also investigated. Results did not show significant differences in clinical outcome related to viral detection. Studies with more statistical power are needed to elucidate the impact of MCPyV DNA on MCC behavior. Half of the cases reported in this study showed MCPyV DNA detection less than 80%, originally reported by Feng and colleagues [5]. The reasons of the lower frequency of MCPyV in MCC could be explained by assuming that our samples included H&N MCCs (38.5%). Our data corroborate results reported in previous studies and showing a lower frequency of MCPyV in H&N MCCs compared to other sites [19,20]. The lower frequency (<80%) of MCPyV-positive MCCs suggests that a proportion of MCC arises through an alternative pathway, possible triggered by UV. Observations suggest that UV sun light long-term exposure is a risk factor for MCC. In particular, UVB index was positively associated with the incidence of MCC in the United States, New Zealand and Australia [21]. In fact, a study on Australian tissue samples from MCC patients reported that only 24% of the MCC contained MCPyV, whereas in northern Europe, 80% of the tumors are associated with MCPyV [21]. This observation suggests that high sun exposure in Australia contributes to the incidence of MCC than the MCPyV [22]. It is tempting to speculate that also in Italy, the latitude, favors to high UV exposure, contributing to the higher incidence of UV-associated MCC than that MCPyV-induced MCC.

Since little is known about MCPyV NCCR and VP1 alterations in the context of MCC and in effort of advancing our understanding of MCPyV biology, positive MCPyV DNA samples were subjected to nested PCR with different MCPyV-specific primer pairs mapping VP1 and NCCR regions of the genome, following published protocols [5,16,17]. Alignment of 13 MCPyV NCCRs (*MCCPL 1–13*) revealed a canonical structure in all analyzed sequences although, compared to the MCC350 [5], some point mutations and insertions were observed. Specifically, a *GTTGA* insertion into nucleotide positions 5210–5211 was observed in *MCCPL 1* and *MCCPL 9*, changing the sequence TATA elements [16] (Table 2). Based on NCCR sequences, Hashida and colleagues identified two subtypes, I and II, with the presence or absence of a 25 base-pair (bp) tandem repeat into nucleotide positions 5177–5178, respectively [23]. Depending on the occurrences of two additional insertions (2 bp, TT, and 5 bp insertions, GTTGA, between nucleotide positions 5199–5200 and 5210–5211, respectively), MCPyV strains were assigned further into five genotypes. Relatively to *MCCPL 1* and *MCCPL 9,* we found their NCCR sequence belonged to the IIa-2 strain, which contains the 5 bp insertion and represents the predominant strain among white persons of European descent [23]. Our results confirm a high degree of sequence stability, suggesting that NCCR rearrangements in this context are probably rare and not involved in the carcinogenesis process. Moreover, it could provide an explanation for the low viral load revealed. In fact, it is well known for other HPyVs that NCCR rearrangements can increase viral replication and influence gene expressions and virulence properties [16]. 

Sequences analysis of MCPyV VP1 amplicons showed some nucleotides differences with respect to the reference strain MCC350 [5], although these variations did not produce any amino-acid change in the derived protein sequence (Table 2). To confirm that MCPyV mutations did not originate from DNA polymerase-induced mistakes, we also amplified and sequenced the NCCR/VP1 of the reference strain MCC350 [5]. Analysis of the sequence revealed that those were identical compared of reference strain, indicating that the PCR did not introduce mutations. The phylogenetic analysis, carried out on the *VP1* gene sequences obtained from MCCPL, showed that the isolates were 99% identical to the reference sequence. All isolates clustered together and with the corresponding reference strain [5]. Since the circulation and the genetic evolution of HPyVs were influenced by virus infectivity and/or virus antigenic variability, monitoring amino-acid changes could be useful to improve the understanding of the epidemiological and clinical features of MCPyV. 

Another interesting observation of this study is the discrepancy between the MCPyV DNA detection in primary and metastatic lesions. In all cases, in which MCCPL and MCCML were investigated, the MCPyV DNA was revealed only in primary lesions. Since previous studies have shown the presence of MCPyV sequences in 46–82% of the examined metastatic lymph nodes [24,25,26], this study represents the first in which none of the metastatic samples tested was PCR positive for MCPyV DNA.

The failure to detect MCPyV DNA in the MCCML samples is certainly not due to poor quality or degradation of DNA in the FFPE metastatic lymph node samples because *beta-globin gene* sequences were amplified, but rather confirm the possibility that in some cases the metastasis lose MCPyV. Our data underscores a “hit-and-run” mechanism in which viral sequences have been lost in the metastatic tumors. The “hit-and-run” theory, also proposed by other authors [27,28], may lead to speculation that in some MCCs the virus is only necessary for the initiation of tumor process and that further mutations may help drive the tumor independent from the virus. Since there is no more selection pressure for maintaining the virus integrated in the genome, new tumor clones may become MCPyV-negative. 

In conclusion, our data suggest that metastasis of primary MCPyV-positive MCCs is associated with loss of the virus. To unequivocally establish a role of MCPyV in malignancies, additional well-controlled investigations are required, and larger cohorts should be examined.

## Figures and Tables

**Table 1:** Detection and quantification of MCPyV DNA by real-time qPCR in MCCPL and MCCML analyzed.

MCCPL Case No.	Age (years)	Gender	Site	qPCR Results
MCCPL 1	77	F	Skin	35 × 10^−1^ copies
MCCPL 2	82	M	forearm	70 × 10^−1^ copies
MCCPL 3	93	F	shoulder	35 × 10^−1^ copies
MCCPL 4	93	F	shoulder	100 × 10^−1^ copies
MCCPL 5	96	F	lower limb	92 × 10^−1^ copies
MCCPL 6	80	M	knee	92 × 10^−1^ copies
MCCPL 7	76	F	forearm	95 × 10^−1^ copies
MCCPL 8	75	M	shinbone	75 × 10^−1^ copies
MCCPL 9	79	M	H&N parotid	95 × 10^−1^ copies
MCCPL 10	92	F	H&N larynx	33 × 10^−1^ copies
MCCPL 11	74	F	H&N eyelid	82 × 10^−1^ copies
MCCPL 12	79	F	H&N superciliar skin	18 × 10^−1^ copies
MCCPL 13	82	M	H&N ear	87 × 10^−1^ copies
MCCPL 14	79	F	shoulder	NEGATIVE
MCCPL 15	76	F	ellipse of skin and subcute arm	NEGATIVE
MCCPL 16	86	F	skin	NEGATIVE
MCCPL 17	84	F	arm	NEGATIVE
**MCCML Case No.**	**Age (years)**	**Gender**	**Site**	**qPCR Results**
MCCML 18	77	F	Inguinal lymph node	NEGATIVE
MCCML 19	82	M	back	NEGATIVE
MCCML 20	79	M	lymph node	NEGATIVE
MCCML 21	79	M	lower limb	NEGATIVE
MCCML 22	79	M	hypocondrium	NEGATIVE
MCCML 23	74	M	inguinal lymph node	NEGATIVE
MCCML 24	93	M	inguinal lymph node	NEGATIVE
MCCML 25	93	M	axillar lymphectomy	NEGATIVE
MCCML 26	85	F	inguinal lymph node	NEGATIVE

MCCPL: primary lesions; MCCML: metastatic lesions; qPCR: quantitative polymerase chain reaction; H&N: head and neck.

**Table 2:** Analysis of VP1 and NCCR sequencing in MCCPL and MCCML analyzed.

MCC Case No.	VP1 Sequencing	NCCR Sequencing	Genotype
MCCPL 1	4192 TΔ	5210–5211 GTTGA ins.	IIa-2 strain
MCCPL 2	4179 CΔ	5220 T to C transition	not applicable
MCCPL 3	no modification	no modification	not applicable
MCCPL 4	4204 T to C transition	no modification	not applicable
MCCPL 5	no modification	no modification	not applicable
MCCPL 6	no modification	5104 G to T transversion	not applicable
MCCPL 7	no modification	no modification	not applicable
MCCPL 8	no modification	no modification	not applicable
MCCPL 9	no modification	5148 T to C transition; 5210–5211 GTTGA ins.	IIa-2 strain
MCCPL 10	4324 A to T transversions	no modification	not applicable
MCCPL 11	no modification	no modification	not applicable
MCCPL 12	no modification	no modification	not applicable
MCCPL 13	no modification	5176 A to T transversions	not applicable

MCCPL: primary lesions; NCCR: non-coding control region; viral capsid protein 1: VP1; Δ: deletion; ins: insertion.

## Data Availability

Data is contained within the article.
